# New Insights Into the Nature of Interspecific Hybrid Sterility in Rice

**DOI:** 10.3389/fpls.2020.555572

**Published:** 2020-09-23

**Authors:** Jing Li, Jiawu Zhou, Yu Zhang, Ying Yang, Qiuhong Pu, Dayun Tao

**Affiliations:** Yunnan Key Laboratory for Rice Genetic Improvement, Food Crops Research Institute, Yunnan Academy of Agricultural Sciences (YAAS), Kunming, China

**Keywords:** allelic variation, hybrid sterility, interspecific hybrid, rice, Oryza sativa

## Abstract

Interspecific and intraspecific hybrid sterility is a typical and common phenomenon of postzygotic reproductive barrier in rice. This is an indicator of speciation involved in the formation of new species or subspecies, and it significantly hampers the utilization of favorable genes from distant parents for rice improvement. The *Oryza* genus includes eight species with the same AA genome and is a model plant for studying the nature of hybrid sterility and its relationship with speciation. Hybrid sterility in rice is mostly controlled by nuclear genes, with more than 50 sterility loci genetically identified to date, of which 10 hybrid sterility loci or pairs were cloned and characterized at the molecular level. Comparing the mapping results for all sterility loci reported indicated that some of these loci from different species should be allelic to each other. Further research revealed that interactions between the multiple alleles at the hybrid sterility locus caused various genetic effect. One hypothesis for this important phenomenon is that the hybrid sterility loci are orthologous loci, which existed in ancient ancestors of rice. When one or more ancestors drifted to different continents, genetic divergence occurred because of adaptation, selection, and isolation among them such that various alleles from orthologous loci emerged over evolutionary time; hence, interspecific hybrid sterility would be mainly controlled by a few orthologous loci with different alleles. This hypothesis was tested and supported by the molecular characterization of hybrid sterility loci from *S1*, *S5*, *Sa*, *qHMS7*, and *S27*. From this, we may further deduce that both allelic and non-allelic interactions among different loci are the major genetic basis for the interspecific hybrid sterility between *O. sativa* and its AA genome relatives, and the same is true for intraspecific hybrid sterility in *O. sativa*. Therefore, it is necessary to raise the near-isogenic lines with various alleles/haplotypes and pyramided different alleles/haplotypes from sterile loci in the same genetic background aiming to study allelic and non-allelic interaction among different hybrid sterility loci in the AA genome species. Furthermore, the pyramiding lines ought to be used as bridge parents to overcome hybrid sterility for rice breeding purposes.

## Introduction

Reproductive barriers are very common and important phenomena in biology, being widely observed in animal and plant populations. Reproductive isolation was classified into pre-zygotic and post-zygotic isolation mechanisms (Smith, 1989). The former prevents the formation of hybrids, and the latter acts after the formation of a hybrid and includes hybrid necrosis, weakness, hybrid sterility, and lethality (Stebbins, 1950).

Hybrid sterility is generally thought to be the most pervasive post-zygotic isolating mechanism, which provides an initial driving force for genetic differentiation and thus plays a key role in speciation (Orr and Presgraves, 2000). One of the major challenges in biology is to understand the origin of species, so the relationship between the hybrid sterility and the formation of new species, or subspecies, is a subject of major interest in evolutionary biology (Darwin, 1859; Coyne and Orr, 2004). Yet, the introgression of favorable genes from distantly relatives through wide crossing and heterosis utilization between subspecies or species are hindered by the hybrid sterility.

Various causes have been ascribed to explain hybrid sterility, such as meiotic irregularities (Yao et al., 1958), chromosomal aberrations (Henderson, 1964), and cytoplasmic-nuclear interactions (Shinjyo, 1975). Recent reports have also speculated that structural variation at particular trait loci may contribute to the intersubspecific hybrid sterility between the *O. sativa* L. ssp. *indica* and *O. sativa* L. ssp. *japonica* accessions (Shen et al., 2017; Wang et al., 2018).

Nevertheless, most known cases of sterility arise from a disharmonious interaction between nuclear genes derived from their respective parents (Stebbins, 1958; Grant, 1981). Genes for hybrid sterility have been reported frequently in fungi, animals, and plants (Brideau et al., 2006; Lee et al., 2008; Bikard et al., 2009). This prominence indicates that gene interactions are critically involved in generating hybrid sterility.

Hybrid sterility occurs widely in hybrids of Asian cultivated rice species, especially between the Asian and African rice species, and between the cultivated rice species and their wild relatives. Because *Oryza* genus had broad genetic diversity and well-characterized genetic base, it is perhaps one of the best model plants to study the nature of hybrid sterility. Based upon published reports of more than 50 sterility loci genetically identified, and 10 hybrid sterility loci or pairs cloned and characterized at the molecular level, this review examines the links between the evolutionary relationship of the *Oryza* genus and hybrid sterility, the genetic models of hybrid sterility, the allelic variation of orthologous loci for hybrid sterility, the non-allelic interactions for hybrid sterility, and strategies for overcoming hybrid sterility in rice improvement.

## Hybrid Sterility and the Classification, Origin, and Evolutionary Relationships of the *Oryza* Genus

The *Oryza* genus has eight AA genome species of diploid chromosome, of which six are wild and two are cultivated (Vaughan, 1989). One of the cultivated species, *O. sativa* L., has a global distribution and is now classified as two subspecies: *O. sativa* L. ssp. *japonica* and *O. sativa* L. ssp. *indica*. The *japonica* subspecies is further classified as three subpopulations, *tropical*, *temperate*, and *aromatic*, while the *indica* subspecies contains two subpopulations, *indica* and *aus* (Garris et al., 2005) or six subpopulations (Wang et al., 2018). Another cultivated species is *O. glaberrima* Steud., which is localized in West Africa and commonly referred to as African rice (Morishima et al., 1963). The six wild species are *O. rufipogon* Griff. and *O. nivara* Sharma et Shastry from Asia; *O. longistaminata* A. Chev. et Roehr. and *O. barthii* A. Chev. from sub-Saharan Africa; *O. meridionalis* Ng from Australia, and *O. glumaepatula* Steud. from South America (Vaughan et al., 2003). Hybrid sterility in rice was frequently observed between and within the AA genome species and was thought to serve as an important indicator for studying these species’ relationships, besides their morphological, physiological, ecological traits, and molecular markers (Kato et al., 1928; Chu et al., 1969; Morishima, 1969; Lu et al., 1998; Naredo et al., 1998; Lu et al., 2000; Vaughan et al., 2003).

The origin and evolutionary relationship of the AA genome nevertheless remain quite contentious, with different opinions and arguments being advanced. Most researchers now believe that the domestication of rice from wild to cultivated species occurred independently in Africa and Asia, respectively (Londo et al., 2006; Vaughan et al., 2008). Recent studies confirmed that *O. glaberrima* was domesticated directly from an *O. barthii* subgroup in a single domestication center along the Niger river (Wang et al., 2014).

One hypothesis is that *japonica* was derived from *indica* (Chang, 1976; Oka, 1988), while according to an alternative hypothesis both *indica* and *japonica* were originated independently from their wild ancestors (Second, 1982; Bautista et al., 2001; Cheng et al., 2003). Based on an analysis of their whole chloroplast genome sequences, the maternal genome of *japonica* may have been derived from *O. rufipogon* and that of *indica* may have originated from *O. nivara*, which would support the independent domestication theory (Wambugu et al., 2015). By employing the *indica-japonica* specific insertion/deletion markers to evaluate the genetic relationships within the genus *Oryza*, Chin et al. (2017) recently provided more evidence of *indica* and *japonica* evolving independently.

All the above studies concerned with rice’s origin and evolutionary history relied on evidence from either archaeological analyses or genetic markers. Given that the strength of reproductive isolation is significantly and positively correlated with parental divergence (Coyne and Orr, 2004), comparative analyses of the timing and evolutionary progression of genetic changes underlying reproductive isolation genes could us better understand the mechanisms of speciation (Moyle and Payseur, 2009). With the advent of molecular biology and in recent years genomic studies especially, the hybrid sterility genes, also called “speciation genes”, provide an unique angle of view for understanding the genetic, origin and evolution of the *Oryza* genus. For instance, the molecular evolution analysis of the *S5* locus, one of the important female sterility loci in *indica*-*japonica* hybrids, revealed that *indica* and *japonica* subspecies of the Asian cultivated rice *O. sativa* were domesticated independently from wild species (Du et al., 2011). This result thus supported the independent origin theory of *indica* and *japonica* and was consistent with the finding reported by Wambugu et al. (2015). The cloning of *S1* locus, which is the most important genetic factor causing the hybrid sterility between two cultivated species, provides experimental evidence for the independent origin of Asian cultivated rice and African cultivated rice (Xie et al., 2019b).

## Genetic Models for the Genetic Mechanism of Hybrid Sterility in Rice

Two genetic models are generally accepted for explaining the genetic mechanism underpinning hybrid sterility in rice according to the Mendelian inheritance: allelic interaction (Kitamura, 1962) and epistatic interaction (Oka, 1957; Oka, 1974). The former proposes that a genetic interaction between two divergent alleles on a single locus causes the abortion of gametes carrying a specific allele, while the latter proposes that epistatic interactions between two loci cause hybrid sterility.

To date, more than 50 hybrid sterility loci in rice have been identified from gene mapping populations generated from various germplasm lines (**Supplementary Table 1**). Some of these loci cause pollen sterility, some cause female gamete abortion, and a few cause the abortion of both. Yet most of these loci seem to act independently on hybrid sterility, which could be explained by the one-locus interaction model; for those showing epistatic interactions, they fit the two-loci interaction model.

From the mapping loci, 10 hybrid sterility loci or pairs of epistatic interaction were cloned and characterized at the molecular level, for which the genetics of seven loci—*S1*, *S5*, *S7*, *Sa*, *Sc*, *hsa1*, and *qHMS7*—follow the one-locus interaction model (Chen et al., 2008; Long et al., 2008; Yang et al., 2012; Kubo et al., 2016; Yu et al., 2016; Shen et al., 2017; Xie et al., 2017; Koide et al., 2018; Yu et al., 2018; Xie et al., 2019b). Most loci contain two or more closely linked genes interacting to cause gamete sterility, which can take these recognized forms: a single Mendelian factor, such as two genes at *Sa*, *qHMS7* and *hsa1* locus (Long et al., 2008; Kubo et al., 2016; Yu et al., 2018); three genes at *S1* and *S5* loci (Yang et al., 2012; Xie et al., 2017; Koide et al., 2018; Xie et al., 2019b); and genomic structural variation at the *Sc* locus (Shen et al., 2017). All can be regarded as interaction between different alleles from a single Mendelian factor to control hybrid sterility.

The other three pairs (*S27/28*, *DPL1/DPL2*, and *DGS1/DGS2*) follow the two-loci interaction model (Mizuta et al., 2010; Yamagata et al., 2010; Nguyen et al., 2017). These gamete-essential genes were interchromosmal duplicated segment from their ancestral loci. The reciprocal loss of one of the duplicated genes caused the sterility in hybridization in the divergent species. Therefore, genetic segregation and recombination gave rise to gametes lacking any duplicated functional genes in the hybrids, thus leading to hybrid sterility (Mizuta et al., 2010; Yamagata et al., 2010; Nguyen et al., 2017).

However, the molecular mechanisms underlying each of the loci are in fact complex though the genetic basis of reproductive isolation seems simple according to classical genetic analysis. Not only are the genes involved in hybrid sterility characterized by very different functional categories, the way in which they interact to cause sterility is also distinct, and this topic has been reviewed before (Ouyang and Zhang, 2013; Xie et al., 2019a). However, it is necessary to recheck hybrid sterility from different perspectives.

## Several Hybrid Sterility Loci From Different Species Should Confer Multiple Alleles

**Figure 1:**
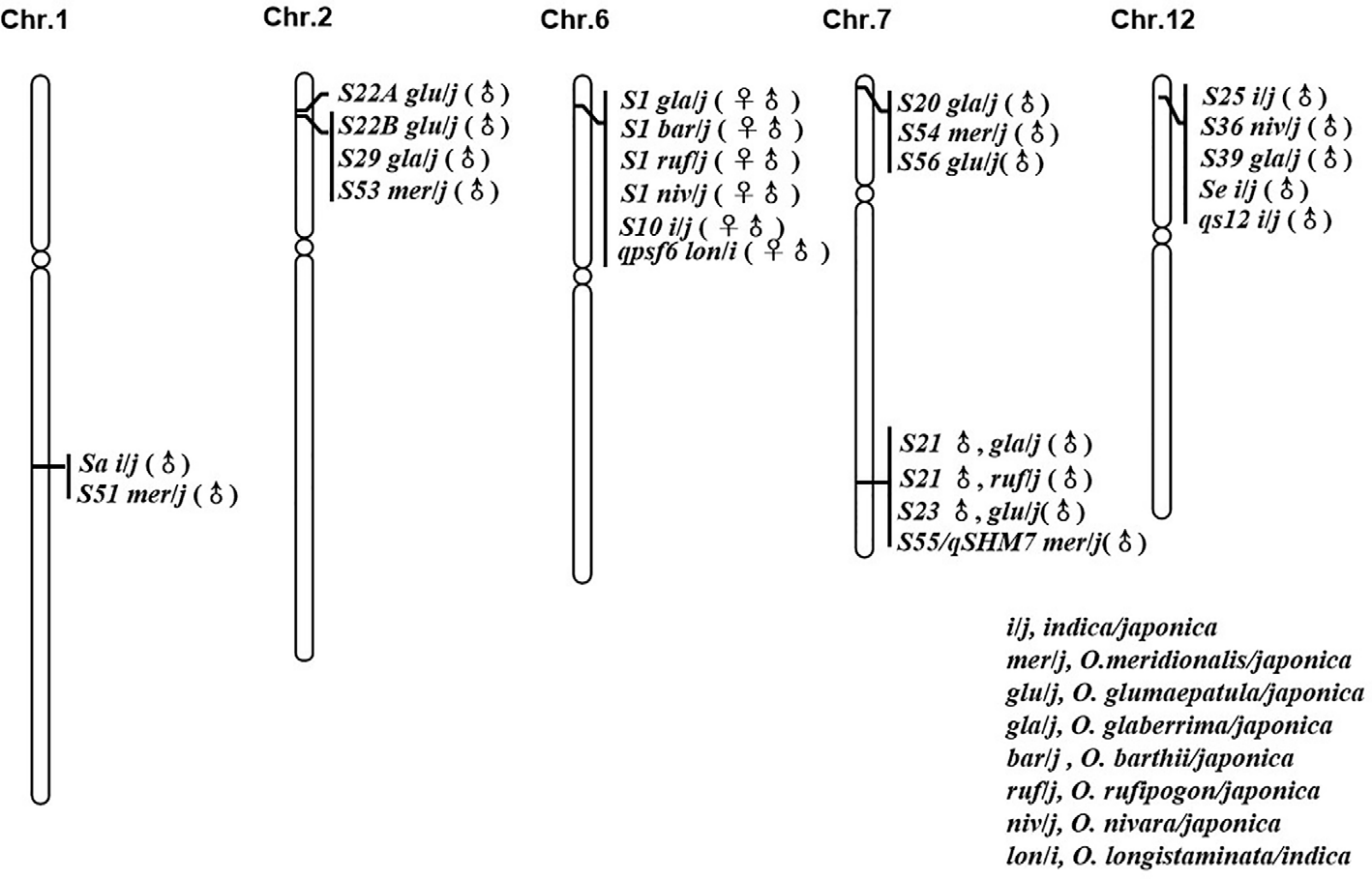
Loci with good co-linear relationship and similar genetic action for hybrid sterility in rice.

In reviewing the progress made in the genetic mapping study of hybrid sterility loci/QTL between *O. sativa* and its AA genome relatives, six “hot spots” from different species were frequently identified (**Figure 1**). Comparing the mapping results for all these sterility loci suggested that some sterility loci arising from multiple species should be allelic to each other, since they were located to the same chromosome region and were identified as having similar genetic activity.

The interspecific hybrid sterility locus *S1* on chromosome 6 was first identified in the cross between *O. sativa* (T65*wx*) and *O. glaberrima* (W025) (Sano et al., 1979). It is frequently reported by a large number of studies between the two cultivated rice species, *O. sativa* (*indica*/*japonica*) and *O. glaberrima* (Koide et al., 2008; Garavito et al., 2010; Zhou et al., 2010; Li J. et al., 2012). The *S1* locus functions as a “gamete eliminator”: both male and female gametes carrying the allele of *O. sativa* are aborted in the heterozygote, for which the type of pollen abortion was of an empty abortion phenotype. The *S10* locus, inducing both male and female gametes’ abortion in an intraspecific hybrid cross between T65*wx* (*japonica* type) and PTB 10 (*indica* type), was mapped onto the similar chromosome position as *S1* on chromosome 6 (Sano et al., 1994), with some later studies indicating that *S1* and *S10* are allelic to each other (Heuer and Miézan, 2003; Zhu et al., 2005). Chen et al. (2009) detected one main-effect QTL, *qpsf6*, for pollen and spikelet fertility on the short arm of chromosome 6 close to the SSR marker RM587, from the cross between an *indica* rice cultivar RD23 of *O. sativa* and an accession of *O. longistaminata*. Comparing their position and effect indicated that this QTL coincides with the gamete eliminator *S1* (Chen et al., 2009). Recently, a genetic study found that *S1* also was a major sterility locus in the hybrid combinations obtained from *O. sativa* crossed with *O. rufipogon*, *O. nivara*, and *O. barthii*, respectively (Yang et al., 2016), which strongly suggests there was an orthologous hybrid sterility locus controlling hybrid sterility between *O. sativa* and its AA genome relatives in this area on rice chromosome 6.

The pollen killer locus *S22*, initially derived from the cross between *O. glumaepatula* and *O. sativa*, was subsequently identified as two closed but linked loci, *S22A* and *S22B.* Since the sterile alleles *S22A* and *S22B* are closely linked and contributed to hybrid sterility in the one-locus allelic interaction model, the *S22A* and *S22B* loci were regarded as a single Mendelian locus in their initial mapping experiments (Sobrizal et al., 2000a; Sakata et al., 2014). A pollen killer locus *S29(t)*, which induced hybrid sterility in the cross between *O. glaberrima* and *O. sativa*, was mapped onto the similar position to *S22* on chromosome 2, whose comparative mapping indicated that *S29(t)* corresponded to *S22B* (Hu et al., 2006; Sakata et al., 2014). *S53*(t), caused pollen sterility in an *O. sativa–O. meridionalis* hybrid, was identified on the same chromosome region harboring *S22B* (Li et al., 2018b). The male gametes carrying the *O. sativa* allele were viable whereas those carrying the *O. glumaepatula/O. meridionalis* allele were aborted in the *S22B* and *S53*(t) loci; hence, these loci from different species should be allelic to each other with multiple alleles. This implies there might be another orthologous hybrid sterility locus between the Asian cultivated rice *O. sativa* and the African rice *O. glaberrima*, and its wild relatives *O. glumaepatula*, *O. meridionalis* on rice chromosome 2.

The major locus *Sa* conferring the *indica-japonica* hybrid male sterility was identified on chromosome 1 with the genetic background of a *japonica* variety (Zhuang et al., 1999). Work by Li et al. (2018b) identified a pollen killer *S51*(t) on chromosome 1 from *O. meridionalis.* The sterile pollen in the heterozygotes of *S51*(t) and *Sa* showed a similarly empty abortion phenotype under I_2_-­KI staining. Additionally, both *S51*(t)^j^ and *Sa* ^j^, the *japonica* alleles, were aborted and were incapable of being transmitted to the progeny *via* the male gametes. According to a comparison of the location and the genetic mode of locus action, the *S51*(t) can be allelic to *Sa* (Li et al., 2018b).

The hybrid sterility locus *S20* was identified on the distal end of the short arm of chromosome 7, from the cross between *japonica* rice (Taichung 65) and African rice Acc. IRGC104038 (Doi et al., 1999). An examination of map positions also revealed that *S20* was highly likely to be allelic to *S54*(t) and *S56*(t), which were respectively identified in the hybridization crossed combinations between *O. sativa* (*japonica*) and *O. meridionalis* (Li et al., 2018b) and between *O. sativa* (*japonica*) and *O. glumaepatula* (Zhang et al., 2018). Just like *S20*, the *japonica* alleles of *S54*(t) and *S56*(t) were aborted and could not be inherited in progeny *via* male gametes, so plants with normal pollen fertility were homozygous for *O. meridionalis* and *O. glumaepatula* allele in the inbreeding population raised from the semi­sterile individuals (Li et al., 2018b; Zhang et al., 2018).

The hybrid sterility locus *S21* was identified on the distal end of the long arm of chromosome 7, from the cross of *japonica* rice (Taichung 65) and African rice Acc. IRGC104038 (Doi et al., 1999). But a new allele of *S21* was identified from *O. rufipogon* Acc. IRGC105715 (Miyazaki et al., 2007). Using the *O. glumaepatula* introgression lines with the background of *O. sativa* (Taichung 65), the pollen semi-sterile locus *S23*(t) was also located on the long arm of chromosome 7 and was thought to be allelic to *S21* from *O. glaberrima* and *O. rufipogon* after comparing their map positions (Sobrizal et al., 2000b; Miyazaki et al., 2007). The pollen killer locus *S55*(t)/*qHMS7*, from *O. meridionalis*, has a map position similar to that of *S21* identified from *O. rufipogon*, *O. glaberrima*, and *S23*(t) identified from *O. glumaepatula* (Doi et al., 1999; Sobrizal et al., 2000b; Miyazaki et al., 2007; Li et al., 2018b; Yu et al., 2018). This congruence pointed to a major pollen sterility locus on the long arm of chromosome 7 that is capable of inducing sterility in the hybridization combinations arising from *O. sativa* crossed with *O. glaberrima*, *O. rufipogon*, *O. glumaepatula*, and *O. meridionalis*, respectively, whose sequence analysis was based on the cloning of *S23* and *qHMS7* (Yu et al., 2018; Fang et al., 2019).

*S3*6 and *S2*5 are two F_1_ pollen sterility loci, both found on the distal end of the short arm of chromosome 12 from the interspecific cross of *O. sativa* (*japonica*) and *O. nivara*, and the intersubspecific cross of *O. sativa*, respectively (Kubo et al., 2001; Win et al., 2009). The pollen sterility locus *Se* and hybrid male sterility QTL *qS12*, both from the intersubspecific cross between *japonica* and *indica*, were mapped onto the short arm of chromosome 12 (Zhu et al., 2008; Zhang H. et al., 2011). Further, the pollen killer *S39*(t) identified from the interspecific cross between *O. sativa* ssp. *japonica* and *O. glaberrima* was likewise mapped onto the same chromosome region of *S36* and *S25* (Xu et al., 2014). Those pollen grains carrying the *japonica* allele were sterile in the heterozygous state. Having a similar map position and same genetic activity, *S25*, *S36*, *S39*, *qS12*, and *Se* might therefore be multiple alleles on the same locus responsible for hybrid sterility in rice.

The six hot spots mentioned above each includes at least two hybrid sterile alleles from different interspecific hybrid crosses, and researchers believe these loci should be allelic to each other, though their allelic relationship has yet to be tested. Nonetheless, the existence of these multiple alleles implies that the hybrid sterility among AA genome species of genus *Oryza* could be governed by a few orthologous loci. It is thus imperative to clarify the allelic relationships of these loci, although any robust confirmation of this allelism is difficult to obtain due to their diverse genetic backgrounds. Based upon this consideration, the near-isogenic lines (NILs) of 5 hybrid sterility loci (*S1*, *S39*, *S44*, *S53*, and *S21*/*qHMS7*) have been developed in the same genetic background, using 35 rice accessions from eight species of AA genome as donors and one *japonica* cultivar (Dianjingyou 1) as the recurrent parent. And the allelic relationship confirmation is ongoing.

## The Allelic Interaction Among Different Alleles of Orthologous Loci and Non-Allelic Interaction Among Different Loci Induce Interspecific Hybrid Sterility Between *O. sativa* and AA Genome Species

One hypothesis for multiple alleles from different species is that these hybrid sterility loci are orthologous loci, which existed in ancient ancestors. When one or more ancestors drifted to different continents, genetic divergence occurred because of adaptation, selection, and isolation among them. With this accumulated genetic differentiation, the various alleles may divide the original function, lose it altogether, or introduce a new function (Lynch and Force, 2000). Hence, interspecific hybrid sterility would be mainly controlled by a few orthologous loci with different alleles/haplotypes. This hypothesis was proven by the molecular characterization of cloned hybrid sterility loci in rice.

The hybrid sterility locus *qHMS7*, which confers pollen semi-sterility in the hybrid between *O. meridionalis* and *O. sativa* ssp. *japonica* Dianjingyou 1 (DJY1), was cloned and molecularly characterized (Yu et al., 2018). The *O. sativa* allele of *ORF2* (*ORF2-D*) and *ORF3* (*ORF3-D*) respectively encoded the toxin and antidote, while *ORF2-mer* was non-functional and *ORF3* was absent in *O. meridionalis*, which induced the pollens carrying the *O. meridionalis* allele to be aborted (Yu et al., 2018). The analyzed gene structure of the *qHMS7* locus indicated that *ORF2* was present in all sequenced accessions, with a total 27 haplotypes of *ORF2* identified, yet only one haplotype of *ORF3* (*ORF3-D*) was identified in parts of *O. rufipogon* and most of Asian-cultivated rice accessions, but none in *O. meridionalis*, *O. longistaminata*, *O. barthii*, and *O. glaberrima* (Yu et al., 2018). So, *qHMS7* was not only detected in the cross of *O. sativa–O. meridionalis* (Yu et al., 2018; Li et al., 2018b) but also in the crosses of *O. sativa–O. glaberrima* and *O. sativa–O. rufipogon* as *S21* (Doi et al., 1999; Miyazaki et al., 2007). The pollen semi-sterility locus *S23* in *O. sativa–O. glumaepatula* has a similar map position to *qHMS7* (Sobrizal et al., 2000b; Fang et al., 2019). Sequence analysis revealed that *S23* was allelic to *qHMS7*, the *ORF2* and *ORF3* of *S23* have the same “Toxin-Antidote” function when compared with *qHMS7*; however, the genetic effect of *S23* differs from *qHMS7* in two ways (Fang et al., 2019). On the one hand, F_1_ pollen fertility was semi-sterility (51.16 ± 1.29%) for *qHMS7* and no homozygote for *O. meridionalis* was obtained, indicating the pollen grains carrying *O. meridionalis* allele had aborted completely; whereas, for *S23*, its F_1_ pollen fertility was higher (63.15 ± 13.49%) than that of *qHMS7* and a few homozygotes of *O. glumaepatula* was found available, indicating that the pollen grains carrying the *O. glumaepatula* allele were not completely sterile (Yu et al., 2018; Fang et al., 2019). On the other hand, the pollen sterility of F_1_ hybrid could only be observed in a short photoperiods, but not in a long photoperiods at the *S23* locus, yet no phenotypic differences were observed in different environments at the *qHMS7* locus (Li et al., 2018b; Yu et al., 2018; Fang et al., 2019). The allele on *O. sativa* interacted with the respective alleles from *O. meridionalis* and *O. glumaepatula*, resulting in hybrid sterility with different genetic effects; accordingly, the allele on *O. glumaepatula* should differ from that on *O. meridionalis*. More research should reveal the nature of interactions occurring between the different haplotypes in rice.

The *Sa* locus was the first cloned and molecularly characterized hybrid sterility locus, from the *indica-japonica* cross, and it comprises two adjacent genes, *SaF* and *SaM* (Long et al., 2008). Most *indica* cultivars have the allele of *SaM* ^+^ *SaF* ^+^, whereas all *japonica* cultivars contain *SaM* ^−^ *SaF* ^−^. The alleles *SaM* ^+^ *SaF* ^+^ and *SaM* ^+^ *SaF* ^−^ were variably present in *Oryza* species having the GG, FF, CC, EE, and AA genomes, including *O. barthii*, *O. nivara*, and *O. sativa* subsp. *indica*. Three alleles in particular *SaM* ^+^ *SaF* ^+^, *SaM* ^+^ *SaF* ^−^, and *SaM* ^−^ *SaF* ^−^ were found in the *O. rufipogon* populations. The mutation of *SaM* most likely arose in an *O. rufipogon* population with the allele *SaM* ^+^ *SaF* ^−^ and then generated the allele *SaM* ^−^ *SaF* ^−^, whereas the variation in *SaF* occurred before the evolutionary split and gave rise to most of the current *Oryza* species (Long et al., 2008). We noticed that eight AA genome species possess the alleles of *SaM* ^+^ *SaF* ^+^ and *SaM* ^+^ *SaF* ^−^, but not *O. sativa* subsp. *japonica*, so it is readily inferred that the hybrid sterility locus *S51*(t) detected from *O. meridionalis–japonica* hybrid was one allele of *Sa* (Li et al., 2018b), with neutral allele *Sa-n* being identified from the *indica* rice and wild rice *O. rufipogon* (Long et al., 2008). It is reasonable to anticipate that, with further research, a tri-allelic system consisting of three types of *Sa* alleles (*SaM* ^+^ *SaF* ^+^, *SaM* ^+^ *SaF* ^−^, and *SaM* ^−^ *SaF* ^−^) will emerge, which is able to control hybrid male sterility and fertility not only in *O. sativa* but also among AA genome species of *Oryza*.

The hybrid sterility locus *S5*, controlling female semi-sterility in hybrids between *indica and japonica*, consists of three closely linked genes *ORF3*, *ORF4*, and *ORF5* that function together in a “Killer–Protector” system (Yang et al., 2012). The typical *indica*-like (*ORF3* ^+^ *ORF4* ^−^ *ORF5* ^+^), *japonica*-like (*ORF3* ^−^ *ORF4* ^+^ *ORF5* ^−^), and neutral (*ORF3* ^+^ *ORF4* ^+^ *ORF5* ^+^ and *ORF3* ^−^ *ORF4* ^−^ *ORF5* ^−^) alleles were found in wild rice accessions of *O. rufipogon* and *O. nivara*; this suggested the *S5* locus already existed in wild relatives and the ancestors of *indica* and *japonica* rice, thus probably originating before domestication (Du et al., 2011; Yang et al., 2012). Presently, *S5* has only been identified in the intersubspecific hybrid cross between *indica* and *japonica*, but according to the distribution of alleles in AA genome we think *S5* also had effect for interspecific hybridization crosses.

Interspecific hybrid sterility locus *S1* is also a tripartite “Killer-Protector” complex (Xie et al., 2019b). The African rice *S1* allele is composed of three adjacent genes (*S1A4*, *S1TPR*, *S1A6*), while the Asian *S1* allele includes only one gene, *S1TP*, which is a truncated form of *S1TPR* (Xie et al., 2017; Koide et al., 2018; Xie et al., 2019). Based on the analyzed gene structure of the *S1* locus, the function structure *A4*-*TPR*-*A6* exists in all examined accessions of *O. glaberrima*; and the nonfunction or dysfunction structureis present in all analyzed accessions of *O. sativa*, *O. longistaminata*, and *O. rufipogon*. The accessions of *O. meridionalis* and *O. barthii* possess both of the structures (Xie et al., 2019b).Total 7 haplotypes and 22 allelic variations of the *S1* locus were detected in the *Oryza* species (Xie et al., 2019b). Further studying the relationship between hybrid sterility and the multiple allelic interactions is needed.

Taken together, the research findings above indicate the interaction between divergent alleles/haplotypes may lead to hybrid sterility, even their interaction mechanisms and genetic effects may be different. It can be further deduced that not only the allelic interactions among different alleles of orthologous loci, but also the non-allelic interactions among different loci, constitutes the major genetic basis for interspecific hybrid sterility between *O. sativa* and other AA genome species.

Benefit from the clone of *S27* and *S28*, a pair of epistatic interaction loci caused F_1_ hybrid sterility in the hybrid pairs *O. sativa–O. glumaepatula* and *O. sativa*–*O. nivara* (Yamagata et al., 2010; Win et al., 2011), we can trace the interaction between different alleles that induce hybrid sterility. The *S27* allele of *O. sativa* is composed of two tandem mitochondrial ribosomal protein L27 genes (*mtRPL27a* and *mtRPL27b*), and the *S28* allele of *O. sativa* contains a loss-of-function allele for *mtRPL27a*. In brief, the *S27* has normal functioning and *S28* has no function in the *O. sativa* allele, whereas only the *S28* has normal functioning and *S27* is absent in the *O. glumaepatula* allele. The epistatic interaction induces the abortion of pollen grains carrying *S27* and *S28*, both of which are inactive in F_1_ progeny of *O. sativa* and *O. glumaepatula*. Judging from a comparison of their genomic sequences, *mtRPL27a* at *S28* on chromosome 4 is thought to be the most ancestral locus. With an interchromosomal duplication, a new copy of *mtRPL27a* at *S27* on chromosome 8 was generated, and likewise *mtRPL27b*, also at *S27* (Ueda et al., 2006). Among the eight AA genome species, the duplicated segment of *S27* was widely observed, occurring in all except *O. glumaepatula*, and only part of the accessions of *O. barthii* and *O. longistaminata* lacked this segment (Yamagata et al., 2010). Further study showed that the interaction between *S27* and *S28* loci also causes the pollen sterility in the F_1_ hybrid of *O. sativa* and *O. nivara*, which inherited the duplicated segment of *S27* (Win et al., 2011). Sequence analysis revealed that the structure of *S27* allele in *O. nivara* is different from that of *O. glumaepatula*; in the former it is composed of two inactive genes (*mtRPL27a*, *mtRPL27b*) while in the latter species these two genes are absent. In short, *S27-niv* is a loss-of-function allele of *S27*. These results highlight that the mechanisms for hybrid sterility are likely different because of epistatic interactions between *S28* loci and different alleles of *S27* in divergent rice species.

The multiple alleles causing hybrid sterility in rice were analyzed at the molecular level for *S5*, *Sa*, *qHMS7/S21/S23/S55*, and *S27*. Meanwhile the cloning of hybrid sterility loci also proves the data support for the allelic relationship of these hybrid sterility loci mentioned above with same molecular location and similar genetic action. According to the latest research, at each hybrid sterility locus, the number of interacting alleles does not seem to be limited to three, of which one is neutral. It has been reported that four alleles characterize the *S7* locus, *S-7* ^kn^, *S-7* ^cp^, *S-7* ^ai^, and *S-7* ^n^, which cause female sterility in the *indica-japonica* hybrid (Yanagihara et al., 1992). The genotype of *S-7* ^ai^/*S-7* ^cp^ shows semi-sterility, in that the female gametes carrying *S-7* ^cp^ are aborted. But in the *S-7* ^ai^/*S-7* ^kn^ genotype, only some of the female gametes possessing *S-7* ^ai^ are aborted. On the other hand, the *S-7* ^ai^/*S-7* ^n^, *S-7* ^n^/*S-7* ^cp^, and*S-7* ^n^/*S-7* ^kn^ genotypes did not exhibit sterility as female gamete abortion (Yanagihara et al., 1992). The initial loci existing in the ancestral species likely differentiated into multiple alleles/haplotypes due to the emergence of genetic differentiation, which accumulated in each population; importantly, this variation was not harmful in the populations in which they arose. Of course, the variation that may affect fitness has been eliminated with the death of the individual. After hybridization between divergence populations, due to the incompatible interaction between two or more functionally diverged genes, the gametes carrying the weak alleles will be aborted, which may be nonfunctional, inactivated, or lack of corresponding protectors. This also explains why the cloned hybrid sterility loci involved in the one-locus systems are mostly composed of more than two linked genes. These closely linked genes, which are distinguished by minimal recombination, form a complex acted as a single Mendelian factor that can avoid inducing the suicidal killer/nonprotector allele in a hybrid that would result in a breakdown of the system (Xie et al., 2019a). The evolutionary history of cloned hybrid sterility genes has been described by many researchers (Long et al., 2008; Yamagata et al., 2010; Du et al., 2011; Kubo et al., 2016; Yu et al., 2018). Ouyang and Zhang (2013) proposed three evolutionary genetic models, those of parallel divergence, sequential divergence, and parallel-sequential divergence, to illustrate the process going from essential genes in the ancestral species to diverged genes based on the cloning data of hybrid sterility genes (Ouyang and Zhang, 2018). By disentangling the interaction between various alleles of these “speciation genes” which underpin the timing and evolutionary progression of genetic changes, we can understand the processes and patterns underlying the speciation and origin of AA genome species of rice further.

## Strategies for Overcoming Hybrid Sterility in Rice Improvement

During the process by which cultivated rice was domesticated from wild ancestor thousands years ago, the cultivated rice’s genetic diversity has been gradually eroded. More importantly, the frequent use of a few elite parents in rice genetic improvement programs has exacerbated this decline in genetic diversity (Simmonds, 1976; Moncada et al., 2001). These challenges hinder our ability to attain further improvement in yield of new varieties while also making rice more susceptible to disease epidemics and pest outbreaks (Tanksley and McCouch, 1997). Thus, the relatives of *O. sativa*, which contain favorable genes (alleles), could be a valuable genetic resource for rice improvement *via* hybridization techniques. On the other hand, the utilization of heterosis in the grain yield of rice has boosted rice production in the past few decades. Current rice hybrids are mostly derived from the *indica* lines. Given their limited genetic diversity, intra-subspecific hybrid vigor now only provides rice breeders with limited yield increases, whereas the distant hybrids derived from intersubspecific even interspecific crosses performed stronger heterosis. However, severe reproductive isolation, such as that incurred by interspecific and intersubspecific hybrid sterility, limits the extensive utilization of these rice distant hybrids (Ikeda et al., 2009; Bolaji and Nwokeocha, 2014). Therefore, hybrid sterility studies should also aim to overcome the reproductive barrier, so as to enable breeders to utilize the valuable extant genetic resources and obtain the strong vigor of these hybrids.

Since hybrid sterility is partly caused by the negative interaction between divergent alleles in the background of the hybrid, replacing the divergent allele with the same or neutral alleles can eliminate the hybrid sterility on the corresponding locus. In this respect, the neutral alleles *S5*-n, *Sa*-n, *Sb*-n, *Sd*-n, and *Se*-n in the intersubspecific hybrid of *indica-japonica* (Ikehashi and Araki, 1986; Long et al., 2008; Li J. Q. et al., 2012), as well as *S38*-n, *S39-*n in the interspecific hybrid of *O. sativa* and *O. glaberrima* (Li et al., 2018a), have been identified. The use of these neutral alleles will give a strong promises on overcoming the interspecific and intersubspecific hybrid sterility. In most cases, as small number of sterility loci are involved in the hybrid cross, so it is necessary to pyramid several sterility loci alleles by using molecular marker assistant backcrossing methods. For example, Guo et al. (2016) polymerized four *indica* alleles of pollen sterility loci (*Sb*, *Sc*, *Sd*, *Se*) and the neutral allele of *S5* locus, imparting the embryo sac sterility to the *japonica* variety, with eight *indica*-compatible *japonica* lines with different *japonica* backgrounds thus obtained. These *indica*-compatible *japonica* lines are highly compatible with the *indica* and effectively overcome the hybrid sterility in the intersubspecific hybrid. In the same vein, two neutral alleles of hybrid sterility loci were introgressed into *indica* rice HJX74 to develop HJX74 wide-compatible lines that were compatible with both *japonica* and *indica* testers (Guo et al., 2016).

Another promising strategy is to create hybrid-compatible lines with artificial neutral alleles generated by advanced molecular approaches, such as RNA interference technologies and gene-editing technology. For example, the neutral allele *S1* ^mut^ was created by mutagenesis and the heterozygous hybrids harboring *S1* ^mut^/*S1* ^g^ and *S1* ^mut^/*S1* ^s^ did not exhibit sterility (Koide et al., 2018). Artificial neutral *S1* alleles could also be obtained by disrupting any one of the three genes in the *S1*-g (*S1A4-S1TPR-S1A6*), through a CRISPP/Cas9 knockout (Xie et al., 2019). The same is true for *qHMS7* (Yu et al., 2018). The introduction of both *Sa*-n and *Sc*-n *via* CRISPR/Cas9 plant genome editing can assist in overcoming unwanted hybrid male sterility (Shen et al., 2017; Xie et al., 2017). A combination of these strategies may provide better assurance of hybrid fertility. With the further discovery and creation of neutral loci and the generation of intersubspecies and interspecies bridge parents, rice improvement will step into a new era.

## Concluding Remarks

The Bateson-Dobzhansky-Muller model posits that hybrid sterility arises from a disharmourose interaction between functionally diverged genes from the hybridizing distant parents (Bateson, 1909; Dobzhansky, 1937; Muller, 1942). This model was first proposed in tomato by Rick (1966) and supported by many other reports (Kitamura, 1962; Ikehashi and Araki, 1986; Sano, 1990). According to the model, independent mutations occur in diverging populations but without a reduction in fitness, becoming fixed; consequently, multiple alleles, including a neutral allele, present at the causal orthologous loci, which then interact negatively in the background of the hybrid (Nei et al., 1983). One locus allelic interaction model underpins the negative interaction occurring at a single locus as a consequence of the independent evolution of two alleles, thereby causing a significant reduction in the fitness of the heterozygote when compared with their homozygote parents. Such negative interaction can also occur between two independent loci affecting gamete development, the gametes carrying the recessive alleles at both loci aborted during gamete development, but gametes of other genotypes remained normal. In this review, we rechecked and updated current genetic models for hybrid sterility in rice. The multiple alleles should exist at each orthologous hybrid sterility locus including, but not limited to, the neutral allele. The major genetic basis for interspecific hybrid sterility between *O. sativa* and AA genome species in genus *Oryza* should be the interaction between various alleles at a single locus, and various alleles with other loci, and the same is true for intersubspecific hybrid sterility in *O. sativa*. Even some of the sterility loci identified in individual combinations/species existed like an orphan, but whether they harbor various alleles in other AA genome species or not requires more study.

Currently, our understanding of hybrid sterility remains limited to that within Asian cultivated rice and between Asian cultivated rice and other species of AA genome, along with a few reports on hybrid sterility among different ecotypes within *O. sativa* that are available. Meanwhile, the availability of information is very limited due to different backgrounds of the genetic materials used by various researchers. To better understand these processes, it is essential to identify, clone, and functionally characterize more hybrid sterility loci in the genus *Oryza*. Efforts should also extend to investigating both the genetic diversity and geographical distribution of the alleles of various loci at the species level, including extant wild relatives. Since allelic and non-allelic interactions among major sterility loci are the foundation for understanding the relationship between hybrid sterility and speciation, it is necessary to raise near-isogenic lines with various alleles/haplotypes and pyramided different loci, with the same genetic background, based on the cloning and molecule characteristics. Then, taking these data together, it would become possible to provide more evidence illuminating the origin of genes responsible for hybrid sterility and the evolutionary processes for the establishment of new species in *Oryza* genus and shed light on overcoming hybrid sterility in rice improvement.

## Author Contributions

DT proposed the insights. JL conceived and wrote the paper. JZ, YZ, YY, and QP reviewed and edited the manuscript.

## Funding

This research was funded by grants from the National Natural Science Foundation of China (grant numbers 31991221, 31860372, and 31660380) and the Key Foundation Project of Yunnan Academy of Agricultural Sciences (grant numberYJZ201801).

## Conflict of Interest

The authors declare that the research was conducted in the absence of any commercial or financial relationships that could be construed as a potential conflict of interest.

The Reviewer YK is currently organizing a Research Topic with one of the authors DT.

## References

[B1] Bateson W. (1909). “Heredity and variation in modern light”. In: Darwin and modern Science. Ed. Seward A. C. (Cambridge: Cambridge Univ. Press), 85–7101.

[B2] Bautista N. S. Solis R. Kamijima O. Ishii T. (2001). RAPD, RFLP and SSLP analyses of phylogenetic relationships between cultivated and wild species of rice. Genes Genet. Syst. 76, 71–79. 10.1266/ggs.76.71 11434461

[B3] Bikard D. Patel D. Le Mette C. Giorgi V. Camilleri C. Bennett M. J. (2009). Divergent evolution of duplicate genes leads to genetic incompatibilities within A. thaliana. Science 323, 623e626. 10.1126/science.1165917 19179528

[B4] Bolaji A. O. Nwokeocha C. C. (2014). Issues concerning reproductive isolation in a rice hybrid swarm involving *Oryza sativa* Linn., *O.* *longistaminata* A. Chev. et Roehr. and *Oryza glaberrima* Steud. located in Jebba Nigeria. Int. J. Biol. Chem. Sci. 7, 2040–2049. 10.4314/ijbcs.v7i5.21

[B5] Brideau N. J. Flores H. A. Wang J. Maheshwari S. Wang X. Barbashi D. A. (2006). Two Dobzhansky-Muller genes interact to cause hybrid lethality in Drosophila. Science 314, 1292–1295. 10.1126/science.1133953 17124320

[B6] Chang T. T. (1976). Origin, evolution, cultivation, dissemination, and Asian and African rice. Euphytica 25, 425–441. 10.1007/BF00041576

[B7] Chen J. Ding J. Ouyang Y. Du H. Yang J. Cheng K. (2008). A triallelic system of *S5* is a major regulator of the reproductive barrier and compatibility of *indica-japonica* hybrids in rice. Proc. Natl. Acad. Sci. U. S. A. 105 (32), 11436–11441. 10.1073/pnas.0804761105 18678896 PMC2516230

[B8] Chen Z. Hu F. Xu P. Li J. Deng X. Zhou J. (2009). QTL analysis for hybrid sterility and plant height in interspecific populations derived from a wild rice relative, *Oryza longistaminata* . Breed. Sci. 59, 441–445. 10.1270/jsbbs.59.441

[B9] Cheng C. Motohashi R. Tsuchimoto S. Fukuta Y. Ohtsubo H. Ohtsubo E. (2003). Polyphyletic origin of cultivated rice: based on the interspersion pattern of SINEs. Mol. Biol. Evol. 20, 67–75. 10.1093/molbev/msg004 12519908

[B10] Chin J. H. Lee Y. J. Jiang W. Koh H. J. Thomson M. J. (2017). Characterization of *indica–japonica *subspecies-specific InDel loci in wild relatives of rice (*Oryza sativa *L. subsp. *indica *kato and subsp. *japonica *kato). Genet. Resour. Crop Evol. 64 (2), 405–418. 10.1007/s10722-016-0368-1

[B11] Chu Y. E. Morishima H. Oka H. II (1969). Reproductive barriers distributed in cultivated rice species and their wild relatives. Jpn. J. Genet. 44, 207–223. 10.1266/jjg.44.207

[B12] Coyne J. A. Orr H. A. (2004). Speciation (Sunderland: Sinauer Associates).

[B13] Darwin C. (1859). On the Origin of Species by Means of Natural Selection or the Preservation of Favoured Races in the Struggle for Life, John Murray. London: Nabu Press. PMC5184128 30164232

[B14] Dobzhansky T. (1937). Genetics and the origin of species (New York: Columbia University Press).

[B15] Doi K. Taguchi K. Yoshimura A. (1999). RFLP mapping of *S20* and *S21* for F1 pollen semi-sterility found in backcross progeny of *Oryza sativa* and *O. glaberrima* . Rice Genet. Newsl. 16, 65–68.

[B16] Du H. Y. Ouyang Y. D. Zhang C. J. Zhang Q. F. (2011). Complex evolution of *S5*, a major reproductive barrier regulator, in the cultivated rice *Oryza sativa* and its wild relatives. New Phytol. 191, 275–287. 10.1111/j.1469-8137.2011.03691.x 21443693

[B17] Fang C. Li L. He R. Wang D. Wang M. Hu Q. (2019). Identification of *S23* causing both interspecific hybrid male sterility and environment-conditioned male sterility in rice. Rice 12, 10. 10.1186/s12284-019-0271-4 30820693 PMC6395467

[B18] Garavito A. Guyot R. Lozano J. Gavory F. Samain S. Panaud O. (2010). A genetic model for the female sterility barrier between Asian and African cultivated rice species. Genetics 185 (4), 1425–1440. 10.1534/genetics.110.116772 20457876 PMC2927767

[B19] Garris A. J. Tai T. H. Coburn J. Kresovich S. Mccouch S. (2005). Genetic structure and diversity in *Oryza sativa* L. Genetics 169, 1631–1638. 10.1534/genetics.104.035642 15654106 PMC1449546

[B20] Grant V. (1981). Plant Speciation (New York: Columbia University Press).

[B21] Guo J. Xu X. Li W. Zhu W. Zhang G. (2016). Overcoming inter-subspecific hybrid sterility in rice by developing *indica*-compatible *japonica* lines. Sci. Rep. 6, 26878. 10.1038/srep26878 27246799 PMC4887987

[B22] Henderson M. T. (1964). Cytogenetic studies at the Louisianan agricultural experiment station on the nature of intervarietal hybrid sterility in Oryza sativa L. [M]// Rice genetics and Cytogenetics (Amsterdom: Elsevier Publishing Co), 147–153.

[B23] Heuer S. Miézan K. M. (2003). Assessing hybrid sterility in *Oryza glaberrima* and*O sativa* hybrid progenies by PCR marker analysis and crossing with wide compatibility varieties. Theor. Appl. Genet. 107, 902–909. 10.1007/s00122-003-1325-x 12851767

[B24] Hu F. Xu P. Deng X. Zhou J. Li J. Tao D. (2006). Molecular mapping of a pollen killer gene *S29*(t) in *Oryza glaberrima* and colinear analysis with *S22* in *O.* *glumaepatula* . Euphytica 151, 273– 278. 10.1007/s10681-006-9146-z

[B25] Ikeda R. Sokei Y. Akintayo I. (2009). Seed fertility of F1 hybrids between upland rice NERICA cultivars and *Oryza sativa* L. or *O. glaberrima* Steud. Breed. Sci. 59, 27–35. 10.1270/jsbbs.59.27

[B26] Ikehashi H. Araki H. (1986). Genetics of F1 sterility in remote crosses of rice, pp. in Rice Genetics. Ed. Khush G. S. (Manila, Philippines: International Rice Research Institute), 119–130.

[B27] Kato S. Kosaka H. Hara S. (1928). On the affinity of rice varieties as shown by fertility of hybrid plants. Bull. Sci. Fac. Agric. Kyushu Univ. 3, 132–147.

[B28] Kitamura E. (1962). Genetic studies on sterility observed in hybrids between distantly related varieties of rice, *Oryza sativa* L. Bull. Chugoku Agric. Exp. Station A8, 141–205.

[B29] Koide Y. Onishi K. Nishimoto D. Baruah A. R. Kanazawa A. Sano Y. (2008). Sex-independent transmission ratio distortion system responsible for reproductive barriers between Asian and African rice species. New Phytol. 179, 888–900. 10.1111/j.1469-8137.2008.02490.x 18507773

[B30] Koide Y. Ogino A. Yoshikawa T. Kitashima Y. Saito N. Kanaoka Y. (2018). Lineage-specific gene acquisition or loss is involved in interspecific hybrid sterility in rice. Proc. Natl. Acad. Sci. U. S. A. 115, E1955–E1962. 10.1073/pnas.1711656115 29444864 PMC5834674

[B31] Kubo T. Eguchi M. Yoshimura A. (2001). A new gene for F1 pollen sterility located on chromosome 12 in *japonica/indica* cross of rice. Rice Genet. Newslett. 18, 54.

[B32] Kubo T. Takashi T. Ashikari M. Yoshimura A. Kurata N. (2016). Two tightly linked genes at the *hsa1* locus cause both F1 and F2 hybrid sterility in rice. Mol. Plant 9, 221–232. 10.1016/j.molp.2015.09.014 26455463

[B33] Lee H. Y. Chou J. Y. Cheong L. Chang N. H. Yang S. Y. Leu J. Y. (2008). Incompatibility of nuclear and mitochondrial genomes causes hybrid sterility between two yeast species. Cell 135 (6), 1065–1073. 10.1016/j.cell.2008.10.047 19070577

[B34] Li J. Q. Shahid M. Q. Feng J. H. Liu X. D. Zhao X. J. Lu Y. G. (2012). Identification of neutral alleles at pollen sterility gene loci of cultivated rice (*Oryza sativa* L.) from wild rice (*O. rufipogon* Griff.). Plant Syst. Evol. 298, 33–42. 10.1007/s00606-011-0520-5

[B35] Li J. Xu P. Zhou J. Hu F. Deng X. Chen Z. (2012). Molecular Mapping of Sterility QTLs *qSS-3*, *qSS-6a* and *qSS-7* as Single Mendelian Factors via NIL strategy. Rice Sci. 18 (2), 110–115. 10.1016/S1672-6308(11)60016-4

[B36] Li J. Zhou J. Xu P. Deng X. Deng W. He M. (2018a). Neutral alleles at hybrid sterility loci of *Oryza glaberrima* from AA genome relatives in *genus Oryza* . Breed. Sci. 68 (3), 343–351. 10.1270/jsbbs.18006 30100801 PMC6081293

[B37] Li J. Zhou J. Xu P. Deng X. Deng W. Zhang Y. (2018b). Mapping five novel interspecific hybrid sterile loci between *Oryza sativa* and *Oryza meridionalis* . Breed. Sci. 68 (5), 516–523. 10.1270/jsbbs.18001 30697112 PMC6345236

[B38] Londo J. P. Chiang Y. C. Hung K. H. Chiang T. Y. Schaal B. A. (2006). Phylogeography of Asian wild rice, *Oryza rufifipogon*, reveals multiple independent domestications of cultivated rice, *Oryza sativa* . Proc. Natl. Acad. Sci. U. S. A. 103, 9578–9583. 10.1073/pnas.0603152103 16766658 PMC1480449

[B39] Long Y. Zhao L. Niu B. Su J. Wu H. Chen Y. (2008). Hybrid male sterility in rice controlled by interaction between divergent alleles of two adjacent genes. Proc. Natl. Acad. Sci. U. S. A. 105 (48), 18871–18876. 10.1073/pnas.0810108105 19033192 PMC2596266

[B40] Lu B. R. Naredo M. E. B. Juliano A. B. Jackson M. T. (1998). Taxonomic status of *Oryza glumaepatula* Steud. III. Assessment of genomic affinity among AA genome species from the New World, Asia, and Australia. Genet. Resour. Crop Evol. 45, 205–214. 10.1023/A:1008686517357

[B41] Lu B. R. Naedo M. E. B. Juliano A. B. Jackson M. T. (2000). “Preliminary studies on taxonomy and biosystematics of the AA genome *Oryza* species (poaceae)” in Grasses, Systematics and Evolution Eds. Jacobs S. W. L. Everett J (CSIRO Publishing), 51–58.

[B42] Lynch M. Force A. G. (2000). The origin of interspecific genomic incompatibility via gene duplication. Am. Nat. 156, 590–605. 10.1086/316992 29592543

[B43] Miyazaki Y. Doi K. Yasui H. (2007). Identification of a new allele of F1 pollen sterility gene, *S21*, detected from the hybrid between *Oryza sativa* and *O. rufipogon* . Rice Genet. Newslett. 23, 36–38.

[B44] Mizuta Y. Harushima Y. Kurata N. (2010). Rice pollen hybrid incompatibility caused by reciprocal gene loss of duplicated genes. Proc. Natl. Acad. Sci. U. S. A. 107, 20417–20422. 10.1073/pnas.1003124107 21048083 PMC2996679

[B45] Moncada P. Martinez C. Borrero J. Chatel M. Gauch H. Jr. Guimaraes E. (2001). Quantitative trait loci for yield and yield components in an Oryza sativa× Oryza rufipogon BC2F2 population evaluated in an upland environment. Theor. Appl. Genet. 102 (1), 41–52. 10.1007/s001220051616

[B46] Morishima H. Hinata K. Oka H. II (1963). Comparison of modes of evolution of cultivated forms from two wild rice species, *Oryza breviligulata* ans *O.* *perennis* . Evolution 17, 170–181. 10.1111/j.1558-5646.1963.tb03267.x

[B47] Morishima H. (1969). Phenetic similarity and phylogenetic relationships among strains of *Oryza* perennis, estimated by methods of numerical taxonomy. Evolution 23, 429–443. 10.2307/2406698 28562931

[B48] Moyle L. C. Payseur B. A. (2009). Reproductive isolation grows on trees. Trends Ecol. Evol. 24 (11), 591–598. 10.1016/j.tree.2009.05.010 19717205

[B49] Muller H. J. (1942). Isolating mechanisms, evolution and temperature. Biol. Symp. 6, 71–125.

[B50] Naredo M. E. B. Juliano A. B. Lu B. R. Jackson M. T. (1998). Taxonomic status of *Oryza glumaepatula* steud. ii. hybridization between new world diploids and aa genome species from asia and australia. Genet. Resour. Crop Evol. 45 (3), 205–214. 10.1023/A:1008634400519

[B51] Nei M. Maruyama T. Wu C. II (1983). Models of evolution of reproductiveisolation. Genetics 103, 557–579. 10.1016/j.acra.2011.04.004 6840540 PMC1202040

[B52] Nguyen G. Yamagata Y. Shigematsu Y. Watanabe M. Miyazaki Y. Doi K. (2017). Duplication and loss of function of genes encoding RNA polymerase III subunit C4 causes hybrid incompatibility in rice. G3 Genes Genomes Genet. 7, 2565–2575. 10.1534/g3.117.043943 PMC5555463 28592558

[B53] Oka H. II (1957). Genic analysis for the sterility of hybrids between distantly related varieties of cultivated rice. J. Genet. 55 (3), 397–409. 10.1007/BF02984059

[B54] Oka H. II (1974). Analysis of genes controlling F1 sterility in rice by the use of isogenic lines. Genetics 77, 521–534. 17248657 10.1093/genetics/77.3.521 PMC1213144

[B55] Oka H. II (1988). Origin of cultivated rice: development in crop species (Tokyo: Jpn Sci Soc Press).

[B56] Orr H. A. Presgraves D. C. (2000). Speciation by postzygotic isolation: forces, genes and molecules. BioEssays 22, 1085–1094. 10.1002/1521-1878(200012)22:12<1085::AID-BIES6>3.0.CO;2-G 11084624

[B57] Ouyang Y. Zhang Q. (2013). Understanding reproductive isolation based on the rice model. Annu. Rev. Plant Biol. 64, 111–135. 10.1146/annurev-arplant-050312-120205 23638826

[B58] Ouyang Y. Zhang Q. (2018). The molecular and evolutionary basis of reproductive isolation in plants. J. Genet. Genomics 45, 613–620. 10.1016/j.jgg.2018.10.004 30459118

[B59] Rick C. M. (1966). Abortion of male and female gametes in the tomato determined by allelic interaction. Genetics 53, 85–96. 10.1016/j.plantsci.2015.09.010 5900610 PMC1211010

[B60] Sakata M. Yamagata Y. Doi K. Yoshimura A. (2014). Two linked genes on rice chromosome 2 for F1 pollen sterility in a hybrid between *Oryza sativa* and *O. glumaepatula* . Breed. Sci. 64, 309–320. 10.1270/jsbbs.64.309 25914585 PMC4267305

[B61] Sano Y. Chu Y. E. Oka H. II (1979). Genetic studies of speciation in cultivated rice, 1. Genic analysis for the F1 sterility between *O. sativa*L. and *O. glaberrima* Steud. Jpn. J. Genet. 54, 121–132. 10.1266/jjg.54.121

[B62] Sano Y. Sano R. Eiguchi M. Hirano H.-Y. (1994). Gamete eliminator adjacent to the *wx* locus as revealed by pollen analysis in rice. J. Heredity 85 (4), 310–312. 10.1093/oxfordjournals.jhered.a111465

[B63] Sano Y. (1990). The genic nature of gamete eliminator in rice. Genetics 125, 183–191. 10.1016/0735-0651(90)90042-E 2341030 PMC1204001

[B64] Second G. (1982). Origin of the genetic diversity of cultivated rice: study of the polymorphism scored at 40 isozyme loci. Jpn. J. Genet. 57, 25–57. 10.1266/jjg.57.25

[B65] Shen R. Wang L. Liu X. Wu J. Jin W. Zhao X. (2017). Genomic structural variation-mediated allelic suppression causes hybrid male sterility in rice. Nat. Commun. 8, 1310. 10.1038/s41467-017-01400-y 29101356 PMC5670240

[B66] Shinjyo C. (1975). Genetical studies of cytoplasmic male sterility and fertility restoration in rice, *Oryza sativa* L. Sci. Bull. Coll. Agri. Univ. Ryukyus 22, 1–57.

[B67] Simmonds N. W. (1976). Evolution of crop plants[M] (London: Longman Group Ltd).

[B68] Smith J. M. (1989). “Macroevolution,” in Evolutionary Genetics (Oxford: Oxford University Press), 272–304.

[B69] Sobrizal Y. M. Sanchez P. Ikeda K. Yoshimura A. (2000a). Identification of a gene for male gamete abortion in backcross progeny of *Oryza sativa* and *Oryza glumaepatula* . Rice Genet. Newslett. 17, 59–61.

[B70] Sobrizal Y. M. Sanchez P. Ikeda K. Yoshimura A. (2000b). Mapping of F1 pollen semi-sterility gene found in backcross progeny of *Oryza sativa* L. and *Oryza glumaepatula* Steud. Rice Genet. Newsl. 17, 61–62.

[B71] Stebbins G. L. (1958). The inviability, weakness, and sterility of interspecific hybrids. Adv. Genet. 9, 147–216. 10.1016/S0065-2660(08)60162-5 13520442

[B72] Stebbins G. (1950). “Isolation and the origin of species,” in Variation and Evolution in Plants (New York: Columbia University Press), 189–250.

[B73] Tanksley S. D. McCouch S. R. (1997). Seed banks and molecular maps: Unlocking genetic potential from the wild. Science 277, 1063–1066. 10.1126/science.277.5329.1063 9262467

[B74] Ueda M. Arimura S. Yamamoto M. P. Takaiwa F. Tsutsumi N. Kadowaki K. (2006). Promoter shuffing at a nuclear gene for mitochondrial RPL27. Involvement of interchromosome and subsequent intrachromosome recombinations. Plant Physiol. 141, 702–710. 10.1104/pp.105.075564 16603668 PMC1475478

[B75] Vaughan D. A. Morishima H. Kadowaki K. (2003). Diversity in the *Oryza* genus. Curr. Opin. Plant Biol. 6 (2), 139–146. 10.1016/S1369-5266(03)00009-8 12667870

[B76] Vaughan D. A. Ge S. Kaga A. Tomooka N. (2008). “Phylogeny and Biogeography of the Genus Oryza,” in Rice Biology in the Genomics Era. Eds. Hirano H.-Y. Hirai A. Sano Y. Sasaki T. (Springer Berlin Heidelberg), 62, 219–234.

[B77] Vaughan D. A. (1989). The genus Oryza L. current status of taxonomy. IRRI Research Paper Series No. 138 (Manila, Philippines: The International Rice Research Institute).

[B78] Wambugu P. W. Brozynska M. Furtado A. Waters D. L. Henry R. J. (2015). Relationships of wild and domesticated rices (*Oryza* AA genome species) based upon whole chloroplast genome sequences. Sci. Rep. 5, 13957. 10.1038/srep13957 26355750 PMC4564799

[B79] Wang M. Yu Y. Haberer G. Marri P. R. Fan C. Goicoechea J. L. (2014). The genome sequence of African rice (*Oryza glaberrima*) and evidence for independent domestication. Nat. Genet. 46 (9), 982–988. 10.1038/ng.3044 25064006 PMC7036042

[B80] Wang W. Mauleon R. Hu Z. Chebotarov D. Tai S. Wu Z. (2018). Genomic variation in 3,010 diverse accessions of Asian cultivated rice. Nature 557, 43–49. 10.1038/s41586-018-0063-9 29695866 PMC6784863

[B81] Win K. T. Kubo T. Miyazaki Y. Doi K. Yamagata Y. Yoshimura A. (2009). Identifification of two loci causing F1 pollen sterility in inter- and intraspecifific crosses of rice. Breed. Sci. 59, 411–418. 10.1270/jsbbs.59.411

[B82] Win K. T. Yamagata Y. Miyazaki Y. Doi K. Yasui H. Yoshimura A. (2011). Independent evolution of a new allele of F1 pollen sterility gene *S27* encoding mitochondrial ribosomal protein L27 in *Oryza nivara* . Theor. Appl. Genet. 122, 385–394. 10.1007/s00122-010-1454-y 20878142

[B83] Xie Y. Xu P. Huang J. Ma S. Xie X. Tao D. (2017). Interspecific hybrid sterility in rice is mediated by *OgTPR1* at the *S1* locus encoding a peptidase-like protein. Mol. Plant 10, 1137– 1140. 10.1016/j.molp.2017.05.005 28533193

[B84] Xie Y. Shen R. Chen L. Liu Y. G. (2019a). Molecular mechanisms of hybrid sterility in rice. Sci. China Life Sci. 62, 737–743. 10.1007/s11427-019-9531-7 31119561

[B85] Xie Y. Tang J. Xie X. Li X. Huang J. Fei Y. (2019b). An asymmetric allelic interaction drives allele transmission bias in interspecific rice hybrids. Nat. Commun. 10, 1–10. 10.1038/S41467-019-10488-3 31175302 PMC6555797

[B86] Xu P. Zhou J. Li F. Hu F. Deng X. Feng S. (2014). Mapping three new interspecific hybrid sterile loci between *Oryzasativa* and *O.* *glaberrima* . Breed. Sci. 63, 476–482. 10.1270/jsbbs.63.476 24757387 PMC3949584

[B87] Yamagata Y. Yamamoto E. Aya K. Win K. T. Doi K. Sobrizal (2010). Mitochondrial gene in the nuclear genome induces reproductive barrier in rice. Proc. Natl. Acad. Sci. U. S. A. 107, 1494–1499. 10.1073/pnas.0908283107 20080642 PMC2824375

[B88] Yanagihara S. Kato H. Ikehashi H. (1992). A new locus for multiple alleles causing hybrid sterility between an *aus* variety and *javanica* varieties in rice (*Oryza sativa* L.). Jpn. J. Breed. 42, 793–801. 10.1270/jsbbs1951.42.793

[B89] Yang J. Zhao X. Cheng K. Du H. Ouyang Y. Chen J. (2012). A killer-protector system regulates both hybrid sterility and segregation distortion in rice. Science 337, 1336e1340. 10.1126/science.1223702 22984070

[B90] Yang Y. Zhou J. Li J. Xu P. Zhang Y. Tao D. (2016). Mapping QTLs for hybrid sterility in three AA genome wild species of *Oryza* . Breed. Sci. 66, 367–371. 10.1270/jsbbs.15048 27436946 PMC4902452

[B91] Yao S. Y. Henderson M. Jodon N. E. (1958). Gryptic structural hybridity as a probable cause of sterility in intervarietal hybrids of cultivated rice, *O. sativa* . Cytologia 23, 46–55. 10.1508/cytologia.23.46

[B92] Yu Y. Zhao Z. Shi Y. Tian H. Liu L. Bian X. (2016). Hybrid sterility in rice (*Oryza sativa* L.) involves the tetratricopeptide repeat domain containing protein. Genetics 203, 1439e1451. 10.1534/genetics.115.183848 27182946 PMC4937479

[B93] Yu X. Zhao Z. Zheng X. Zhou J. Kong W. Wang P. (2018). A selfifish genetic element confers non-Mendelian inheritance in rice. Science 360, 1130e1132. 10.1126/science.aar4279 29880691

[B94] Zhang Y. Zhao Z. Zhou J. Jiang L. Bian X. Wang Y. (2011). Fine mapping of a gene responsible for pollen semi-sterility in hybrids between *Oryza sativa* L. and *O. glaberrima* Steud. Mol. Breed. 28, 323–334. 10.1007/s11032-010-9485-2

[B95] Zhang Y. Zhou J. Li J. Yang Y. Xu P. Tao D. (2018). Mapping of *S56*(t) responsible for interspecific hybrid sterility between *Oryza sativa* and *Oryza glumaepatula* . Breed. Sci. 68, 242–247. 10.1270/jsbbs.17116 29875608 PMC5982189

[B96] Zhou J. Xu P. Deng X. Li J. Hu F. Ren G. (2010). Genetic dissection of a chromosomal region conferring hybrid sterility using multi-donors from *Oryza glaberrima* . Euphytica 175 (3), 395–407. 10.1007/s10681-010-0188-x

[B97] Zhu S. Jiang L. Wang C. Zhai H. Li D. Wan J. (2005). The origin of a weedy rice ludao in China deduced by a genome wide analysis of its hybrid sterility genes. Breed. Sci. 55, 409–414. 10.1270/jsbbs.55.409

[B98] Zhu W. Li W. Ding X. Zhang Z. Zeng R. Zhu H. (2008). Preliminary identification of F1 pollen sterility gene *S-e* in *Oryza sativa* . J. South. China Agricult. Univ. 29, 1–5.

[B99] Zhuang C. Zhang G. Mei M. Lu Y. (1999). Molecular mapping of the *S-a* locus for F1 pollen sterility in cultivatedrice (*Oryza sativa* L.). Acta Genet. Sin. 26, 213–218. 10589160

