# Supplementary material for: New Insights Into the Nature of Interspecific Hybrid Sterility in Rice
Source: Front Plant Sci. 2020 Sep 23;11:555572. doi: 10.3389/fpls.2020.555572 (PMC7538986; doi:10.3389/fpls.2020.555572)
Supplement: Supplementary file 1 [file Table_1.docx]

Table S1 Delimitation of hybrid sterility gene in rice

| Loci | Chr. | Aborting gamete | Cross and Aborting Action | Reference |
| --- | --- | --- | --- | --- |
| *S1* | 6 | ***♂/♀*** | *O. glaberrima → O. sativa* | Sano *et al*. 1979  Koide *et al.*. 2018  Xie *et al.*. 2017, 2019 |
|  |  |  | *O. longistaminata → O. sativa* | Chen *et al.*. 2009 |
|  |  |  | *O. nivara → O. sativa* | Yang *et al.*. 2016 |
|  |  |  | *O. barthii → O. sativa* |  |
|  |  |  | *O. rufipogon→ O. sativa* |  |
| *S2* |  | ♂/♀ |  | Sano *et al.*. 1979 |
| *S3* | 9 | ♂ | *O. sativa → O. glaberrima* | Sano 1983 |
| *S5* | 6 | ♀ | *Indica → Japonica* | Ikehashi and Araki 1986, Chen *et al.*. 2008, Yang *et al.*. 2012 |
| *S6* | 6 | ♂/♀ | *O. rufipogon → O. sativa* | Sano 1989 |
| *S-7* | 7 | ♀ | *indica → japonica* | Yanagihara *et al.*. 1992, Yu *et al.*. 2016 |
| *S-8* | 6 | ♀ | *indica → japonica* | Wan *et al.*. 1993 |
| *S-9* | 4 | ♀ | *indica → japonica* | Wan *et al.*. 1996 |
| *S10* | 6 | ♂/♀ | *indica → japonica* | Sano *et al.* 1994 |
| *S11*(t) | 11 | ♂ | *japonica → indica* | Sawamura and Sano 1996 |
| *S12*(t) |  | ♂ | *O. glumaepatula → O. sativa* | Sano 1994 |
| *S13* | 1 | ♂ |  | Koide *et al*. 2007 |
| *S14*(t) |  | ♂ |  | Sano 1994 |
| *S15* | 12 | ♀ | *indica → japonica* | Wan *et al.*. 1996 |
| *S16* | 1 | ♀ | *japonica → indica* | Wan and Ikehashi. 1995 |
| *S17* | 12 | ♀ | *japonica → indica* | Wan *et al.*. 1998 |
| *S18* | 10 | ♂ |  | Doi *et al.*. 1998 |
| *S19* | 3 | ♂ | *O. glaberrima → O. sativa* | Taguchi *et al.*. 1999 |
| *S20* | 7 | ♂ | *O. glaberrima → O. sativa* | Doi *et al.*. 1999 |
| *S21* | 7 | ♂ | *O. sativa → O. glaberrima* | Doi *et al.*. 1999 |
|  | 7 | ♂ | *O. sativa → O. rufipogon* | Miyazaki *et al.*. 2007 |
| S22A and S22B | 2 | ♂ | *O. sativa → O. glumaepatula* | Sobrizal *et al.*. 2000a,  Sakata *et al.* 2014 |
| *S23*(t) | 7 | ♂ | *O. sativa → O. glumaepatula* | Sobrizal *et al.*. 2000b  Fang *et al.*. 2019 |
| *S24*(t) | 5 | ♂ | *japonica → indica* | Kubo *et al.*. 2000 |
| *S25*(t) | 12 | ♂ | *japonica → indica* | Kubo *et al.*. 2001 |
| *S26*(t) | 6 | ♀ | *japonica → indica* | Kubo and Yoshimura.. 2001 |
| *S27* | 8 | ♂ | *O. sativa → O. glumaepatula* | Sobrizal and Yoshimura 2001, Yamagata *et al.*. 2010 |
|  | 8 | ♂ | *O. sativa → O. nivara* | Win *et al.*. 2011 |
| *S28* | 4 | ♂ | *O. glumaepatula → O. sativa* | Sobrizal and Yoshimura 2002, Yamagata *et al.*. 2010 |
| *S29*(t) | 2 | ♂ | *O. glaberrima → O. sativa* | Hu *et al.*. 2004 |
| *S29*(t) | 2 | ♀ | *japonica → indica* | Zhu *et al.*. 2005b |
| *S30*(t) | 7 | ♀ | *indica → japonica* | Zhu *et al.*. 2005a |
| *S31*(t) | 5 | ♀ | *indica → japonica* | Zhao *et al.*. 2006 |
| *S32*(t) | 2 | ♀ | *indica → japonica* | Li *et al.*. 2005 |
| *S33*(t) | 1 | ♂/♀ | *O. glaberrima → O. sativa* | Ren *et al.*. 2005 |
| *S33*(t) | 3 | ♂ | *japonica → indica* | Jing *et al.*. 2007 |
| *S34*(t) | 11 | ♂ | *japonica → indica* |  |
| *S34*(t) | 3 | ♂ | *O. glaberrima → O. sativa* | Zhang *et al.*. 2005 |
| *S35*(t) | 12 | ♀ | *indica → japonica* | Chen *et al.*. 2012 |
| *S35* | 1 | ♂ | *japonica → indica* | Kubo *et al.*. 2008 |
| *S36* | 12 | ♂ | *O.nivara → O. sativa* | Win *et al.*. 2009 |
| *S37* | 1 | ♂/♀ | *O. glaberrima → O. sativa* | Xu *et al.*. 2014 |
| *S38* | 4 | ♂ | *O. glaberrima → O. sativa* |  |
| *S39* | 12 | ♂ | *O. glaberrima → O. sativa* |  |
| *S40* | 1 | ♂/♀ | *O. longistaminata → O. sativa* | Chen *et al.*. 2017 |
| *S44* | 6 | ♂ | *O. longistaminata → O. sativa* | Zhao *et al.*. 2012 |
| *S51* | 1 | ♂ | *O.meridionalis → O. sativa* | Li *et al.*. 2018 |
| *S52* | 2 | ♂ | *O. sativa → O.meridionalis* |  |
| *S53* | 2 | ♂ | *O. sativa → O.meridionalis* |  |
| *S54* | 7 | ♂ | *O.meridionalis → O. sativa* |  |
| *S55/qHMS7* | 7 | ♂ | *O. sativa → O.meridionalis* | Li *et al.*. 2018, Yu *et al.*. 2018 |
| *S56* | 7 | ♂ | *O. glumaepatula → O. sativa* | Zhang *et al.*. 2018 |
| *hsa1* | 12 | ♀ |  | Kubo *et al.*. 2016 |
| *Sa* | 1 | ♂ | *indica → japonica* | Zhuang *et al.*. 1999, Long *et al.*. 2008 |
| *Sb* | 5 | ♂ | *indica → japonica* | Li *et al.*. 2006 |
| *Sc* | 3 | ♂ | *indica → japonica* | Zhang *et al*. 2001, Shen *et al.*. 2017 |
| *Sd* | 1 | ♂ | *indica → japonica* | Zhang *et al.*. 1994 |
| *Se* | 12 | ♂ | *indica → japonica* |  |
| *Sf* |  | ♂ | *indica → japonica* |  |
| *S-p* | 11 | ♀ | *indica → japonica* | Zhu *et al.*. 1998 |
| *DPL1* | 1 | ♂ |  | Mizuta *et al.*. 2010, |
| *DPL2* | 6 |  |  |  |
| *DGS1* | 4 | ♂ |  | Nguyen *et al.*. 2017 |
| *DGS2* | 7 |  |  |  |

References

Chen, H., Zhao, Z., Liu, L., Kong, W., Lin, Y., You, S., *et al*. (2017). Genetic analysis of a hybrid sterility gene that causes both pollen and embryo sac sterility in hybrids between *Oryza sativa* L. and *Oryza longistaminata*. Heredity, 119: 1-8.

Chen, J., Ding, J., Ouyang, Y., Du, H., Yang, J., Cheng, K.*et al.* (2008). A triallelic system of *S5* is a major regulator of the reproductive barrier and compatibility of *indica-japonica* hybrids in rice. Proc Natl Acad Sci USA, 105(32), 11436-11441.

Chen, M., Zhao, Z., Jiang, L., Wan, J. (2012). A new gene controlling hybrid sterility in rice (*Oryza sativa* L.). Euphytica, 184: 15-22

Chen, Z., Hu, F., Xu, P., Li, J., Deng, X., Zhou, J. *et al*. (2009). QTL analysis for hybrid sterility and plant height in interspecific populations derived from a wild rice relative, *Oryza longistaminata*. Breeding Science, 59: 441-445

Doi, K., K.Tagushi and A.Yoshimura (1998) A new locus affecting high F1 pollen sterility found in backcross progenies of *japonica* rice and African rice. Rice Genet. Newsl. 15: 146–148.

Doi, K., Taguchi, K.. Yoshimura, A. (1999) RFLP mapping of *S20* and *S21* for F1 pollen semi-sterility found in backcross progeny of *Oryza sativa* and *O. glaberrima*. Rice Genet. Newsl. 16: 65–68.

Fang, C., Li, L., He, R., Wang, D., Wang, M., Hu, Q., *et al*.(2019). Identification of *S23* causing both interspecific hybrid male sterility and environment-conditioned male sterility in rice. Rice, 12: 10

Hu, F., Xu, P., Deng,X., Zhou,J., Li, J. and Tao, D. (2006). Molecular mapping of a pollen killer gene *S29*(t) in *Oryza glaberrima* and colinear analysis with *S22* in *O. glumaepatula*. Euphytica 151: 273– 278.

Ikehashi, H. & Araki, H., (1986). Genetics of F1 sterility in remote crosses of rice, pp. in Rice Genetics, edited by G. S. KHUSH. International Rice Research Institute, 119-130

Jing, W., Zhang, W., Jiang, L., Chen, L., Zhai, H., Wan, J. (2007). Two novel loci for pollen sterility in hybrids between the weedy strain ludao and the *japonica* variety akihikari of rice (*Oryza sativa* l.). Theor Appl Genet, 114(5), 915-925.

Koide, Y. , Ikegaya, T. , Nishimoto, D. , Kanazawa, A. , & Sano, Y. . (2007). Hybrid male sterility induced by *S13* found in a distantly related rice species. Genes & Genetic Systems, 82(6), 522-522.

Koide, Y., Ogino, A., Yoshikawa, T., Kitashima, Y., Saito, N., Kanaoka, Y., *et al.* (2018). Lineage-specific gene acquisition or loss is involved in interspecific hybrid sterility in rice. Proc Natl Acad Sci USA 115, E1955–E1962.

Kubo, T., Eguchi, M., Yoshimura, A. (2000). A new gene for F1 pollen sterility in *japonica/indica* cross of rice. Rice Genetics Newsletter, 17: 63-64.

Kubo, T., Eguchi, M., Yoshimura, A. (2001) A new gene for F1 pollen sterility located on chromosome 12 in *japonica/indica* cross of rice. Rice Genetics Newsletter, 18: 54.

Kubo, T., Takashi, T., Ashikari, M., Yoshimura, A., Kurata, N. (2016) Two tightly linked genes at the *hsa1* locus cause both F1 and F2 hybrid sterility in rice. Molecular plant, 9: 221~232

Kubo, T., Yamagata, Y., Eguchi, M., Yoshimura, A. (2008) A novel epistatic interaction at two loci causing hybrid male sterility in an inter-subspecific cross of rice (*Oryza sativa* L.). Genes Genet. Syst. 83: 443-453

Kubo, T., Yoshimura, A. (2001) Linkage analysis of an F1 sterility gene in *japonica*/*indica* cross of rice. Rice Genetics Newsletter,18, 52–54.

Li, D., Zhu, S., Jiang, L. (2005). Mapping for a new locus causing hybrid sterility in a China landrace (*Oryza sativa* L.). Rice Genetics Newsletter, 22: 17-19.

Li, J., Zhou, J., Xu, P., Deng, X., Deng, W., Zhang, Y., *et al*. (2018) Mapping five novel interspecific hybrid sterile loci between *Oryza sativa* and *Oryza meridionalis*. Breeding Science, 68(5): 516-523

Li, W., Zeng, R., Zhang, Z., Ding, X. & Zhang, G. (2006). Fine mapping of locus S-b for F1 pollen sterility in rice(*Oryza sativa* L.). Chin. *Sc*i. Bull. 51, 675–680 .

Long, Y., Zhao, L., Niu, B., Su,J., Wu, H., Chen, Y. *et al.* (2008) Hybrid male sterility in rice controlled by interaction between divergent alleles of two adjacent genes. Proc Natl Acad Sci U S A, 105(48), 18871-18876.

Miyazaki, Y., Doi, K., Yasui, H. (2007) Identification of a new allele of F1 pollen sterility gene, *S21*, detected from the hybrid between *Oryza sativa* and *O. rufipogon*. Rice Genetics Newsletter, 23: 36~38

Mizuta, Y., Harushima, Y., and Kurata, N. (2010). Rice pollen hybrid incompatibility caused by reciprocal gene loss of duplicated genes. Proc Natl Acad Sci USA 107, 20417–20422.

Nguyen G, Yamagata Y, Shigematsu Y, Watanabe M, Miyazaki Y, Doi K, *et al*. (2017) Duplication and loss of function of genes encoding RNA polymerase III subunit C4 causes hybrid incompatibility in rice. G3 Genes Genomes Genetics, 7: 2565~2575

Ren, G., Xu, P., Deng, X., Zhou, J., Hu, F., Li, J., *et al.*(2005) A new gamete eliminator from *Oryza glaberrima.* Rice Genetics Newsletter, 22: 45-47.

Sakata,M., Y.Yamagata, K.Doi and A.Yoshimura (2014) Two linked genes on rice chromosome 2 for F1 pollen sterility in a hybrid between *Oryza sativa* and *O. glumaepatula*. Breeding Science 64: 309–320.

Sano, Y. (1983) A new gene controlling sterility in F1 hybrids of two cultivated rice species. Journal of Heredity, 74: 435-439.

Sano, Y. (1989) Gamete eliminator detected in the wild progenitor of *Oryza sativa*. Rice Genetics Newsletter, 6: 93-94.

Sano, Y. (1994a) Pollen-killers in rice. Japanese Journal of Breeding, 44: 298.

Sano, Y., Chu, Y. E., Oka, H. I. (1979) Genetic studies of speciation in cultivated rice, 1. Genic analysis for the F1 sterility between *O. sativa*L. and *O. glaberrima* Steud. Jpn J Genet 54:121–132.

Sawamura, N., Sano, Y. (1996) Chromosomal location of gamete eliminator, *S11*(t), found in an *indica-japonica* hybrid. Rice Genet Newsletter, 13: 70-71

Shen, R., Wang, L., Liu, X., Wu, J., Jin, W., Zhao, X., *et al*. (2017). Genomic structural variation-mediated allelic suppression causes hybrid male sterility in rice. Nat. Commun. 8, 1310.

Sobrizal, Y. M., Sanchez, P., Ikeda, K., Yoshimura, A. (2000a) Identification of a gene for male gamete abortion in backcross progeny of *Oryza sativa* and *Oryza glumaepatula*. Rice Genetics Newsletter, 17: 59-61.

Sobrizal, Y. M., Sanchez, P., Ikeda, K., Yoshimura, A. (2000b) Mapping of F1 pollen semi-sterility gene found in backcross progeny of *Oryza sativa* L. and *Oryza glumaepatula* Steud. Rice Genetics Newsletters, 17: 61-62

Sobrizal, Y. M., Yoshimura, A. (2001). Mapping of a gene for pollen semi-sterility on chromosome 8 of rice. Rice Genetics Newsletter, 18: 59-61.

Sobrizal, Y. M., Yoshimura, A. (2002) Mapping of pollen semi-sterility gene, *S28*(t), on rice chromosome 4. Rice Genetics Newsletter, 19: 80-81.

Taguchi, K., Doi, K., Yoshimura, A. (1999) RFLP mapping of *S19*, a gene for F1 pollen semi-sterility found in backcross progeny of *Oryza sativa* and *O. glaberrima*. Rice Genetics Newsletter, 16: 70-71.

Wan, J., Ikehashi, H. (1995) Identification of a new locus *S-16* causing hybrid sterility in native rice varieties (*Oryza sativa* L.) from Tai-hu Lake region and Yunnan province, China. Breeding Science, 45: 461-470.

Wan, J., Ikehashi, H., Sakai, M., Horisue, H., Imbe, T. (1998) Mapping of hybrid sterility gene *S17* of rice (*Oryza sativa* L.) by isozyme and RFLP markers. Rice Genetics Newsletter, 15: 151-154.

Wan, J., Yamaguchi, Y., Kato, H., Ikehashi, H. (1996) Two new loci for hybrid sterility in cultivated rice (*Oryzasativa* L.). Theor Appl Genet, 92: 183-190.

Wan, J., Yanagihara, S., Kato, H., Ikehashi, H.(1993) Multiple alleles at a new locus causing hybrid sterility between a Korean *indica* variety and a *japonica* variety in rice. Japanese Journal of Breeding, 43(4): 507-516, doi: 10.1270/jsbbs1951.43.507

Win, K.T., Kubo, T., Miyazaki, Y., Doi, K., Yamagata, Y., Yoshimura, A. (2009). Identifification of two loci causing F1 pollen sterility in inter- and intraspecifific crosses of rice. Breeding Science 59:411–418

Win, K.T., Yamagata, Y., Miyazaki, Y., Doi, K., Yasui, H. and Yoshimura, A. (2011) Independent evolution of a new allele of F1 pollen sterility gene *S27* encoding mitochondrial ribosomal protein L27 in *Oryza nivara*. Theor. Appl. Genet. 122: 385–394.

Xie, Y., Tang, J., Xie, X., Li, X., Huang, J., Fei, Y., *et al.* (2019). An asymmetric allelic interaction drives allele transmission bias in interspecific rice hybrids. Nat Commun, 10:1-10, doi: 10.1038/S41467-019-10488-3.

Xie, Y., Xu, P., Huang, J., Ma, S., Xie, X., Tao, D.*,et al*. (2017). Interspecific hybrid sterility in rice is mediated by *OgTPR1* at the *S1* locus encoding a peptidase-like protein. Mol Plant 10, 1137– 1140.

Xu, P., Zhou, J., Li, F., Hu, F., Deng, X., Feng, S., *et al*. (2014) Mapping three new interspecific hybrid sterile loci between *Oryzasativa* and *O. glaberrima*. Breeding Science, 63: 476–482.

Yamagata, Y., Yamamoto, E., Aya, K., Win, K. T., Doi, K., Sobrizal, *et al.* (2010). Mitochondrial gene in the nuclear genome induces reproductive barrier in rice. Proc Natl Acad Sci USA 107, 1494–1499.

Yanagihara, S., Kato H., and Ikehashi H., (1992) A new locus for multiple alleles causing hybrid sterility between an *aus* variety and *javanica* varieties in rice (*Oryza sativa* L.). Japanese Journal of Breeding 42: 793–801；

Yang, J., Zhao, X., Cheng, K., Du, H., Ouyang, Y., Chen, J., *et al*. (2012). A killer-protector system regulates both hybrid sterility and segregation distortion in rice. Science 337, 1336e1340.

Yang, Y., Zhou, J., Li, J., Xu, P., Zhang, Yu., Tao, D.. (2016). Mapping QTLs for hybrid sterility in three AA genome wild species of *Oryza*. Breeding Science 66: 367–371.

Yu, X., Zhao, Z., Zheng, X., Zhou, J., Kong, W., Wang, P., *et al*. (2018). A selfifish genetic element confers non-Mendelian inheritance in rice. Science 360, 1130e1132.

Yu, Y., Zhao, Z., Shi, Y., Tian, H., Liu, L., Bian, X., *et al*. (2016). Hybrid sterility in rice (*Oryza sativa* L.) involves the tetratricopeptide repeat domain containing protein. Genetics 203, 1439e1451.

Zhang, G., Lu, Y., Zhang, H., Yang, J., Liu, G. (1994). Genetic studies on the hybrid sterility in culitivated rice (*Oryza sativa*) IV Genotypes for F_1 Pollen Sterility. Acta Genetica Sinica, 21 (1): 34-41.

Zhang, Y., Zhou, J., Li, J., Yang, Y., Xu, P., Tao, D. (2018) Mapping of *S56*(t) responsible for interspecific hybrid sterility between *Oryza sativa* and *Oryza glumaepatula*. Breeding Science, 68: 242–247

Zhang, Z. & Zhang, G. (2001). Fine mapping of the *S-c* locus and marker-assisted selection using PCR markers inrice. Acta Agronomica Sinica 27, 704–709. doi: 10.4028/www.scientific.net/MSF.821-823.77

Zhang, Z., Xu, P., Hu, F., Zhou, J., Li, J., Deng, X., *et al*. (2005). A new sterile gene from *Oryza glaberrima* on chromosome 3. Rice Genetics Newsletter, 22: 26-28.

Zhao, J., Li, J., Xu, P., Zhou, J., Hu, F., Deng, X.,et al. (2012) A new gene controlling hybrid sterility between *Oryza sativa* and *Oryza longistaminata.* Euphytica, 187: 339–344

Zhao, Z., Wang, C., Jiang, L., Zhu, S., Ikehashi, H., & Wan, J. (2006). Identification of a new hybrid sterility gene in rice (*Oryza sativa* L.). Euphytica, 151(3), 331-337.

Zhu, S., Jiang, L.，Wang, C., Zhai, H., Li, D., Wan, J. (2005a) The origin of a weedy rice ludao in China deduced by a genome wide analysis of its hybrid sterility genes. Breeding Science, 55: 409-414

Zhu, S., Wang, C., Zheng, T., Zhao, Z., Ikehashi, H., Wan, J. (2005b). A new gene located on chromosome 2 causing hybrid sterility in a remote cross of rice. Plant Breeding,124: 440-445.

Zhu, X., Wang, J., Qian, Q., Zhang, X., Zeng, D., & Zhu L., *et al* (1998). Genetic analysis on a new sterile locus discovered in hybrids between *indica* and *japonica* rice (*Oryza sativa* l.)]. Journal of Genetics & Genomics, 25(3), 245-251. doi: http://dx.doi.org/

Zhuang, C., Zhang, G., Mei, M. & Lu, Y. (1999). Molecular mapping of the *S-a* locus for F1 pollen sterility in cultivatedrice (*Oryza sativa* L.). Acta Genet. Sin. 26, 213–218.
