# Supplementary material for: New Insights Into the Nature of Interspecific Hybrid Sterility in Rice
Source: Front Plant Sci. 2020 Sep 23;11:555572. doi: 10.3389/fpls.2020.555572 (PMC7538986; doi:10.3389/fpls.2020.555572)
Supplement: Supplementary file 2 [file Table_2.docx]

Table S1 Delimitation of hybrid sterility gene in rice

| Loci | Chr. | Genomic location | Aborting gamete | Cross and Aborting Action | Reference |
| --- | --- | --- | --- | --- | --- |
| *S1/qpsf6* | 6 | RM19357-RMC6_22028 | ***♂/♀*** | *O. glaberrima → O. sativa* | Sano *et al*. 1979  Koide *et al.*. 2018  Xie *et al.*. 2017b, 2019b |
|  |  | RM190-RM587 |  | *O. longistaminata → O. sativa* | Chen *et al.*. 2009 |
|  |  | RM190-RM510 |  | *O. nivara → O. sativa* | Yang *et al.*. 2016 |
|  |  | RM190-RM587 |  | *O. barthii → O. sativa* |  |
|  |  | RM190-RM3414 |  | *O. rufipogon→ O. sativa* |  |
| *S2* |  |  | ♂/♀ | *O. sativa / O. glaberrima* | Sano *et al.*. 1979 |
| *S3* | 9 | linked with *la* gene | ♂ | *O. sativa → O. glaberrima* | Sano 1983 |
| *S5* | 6 | RG213-Est2 | ♀ | *indica → japonica* | Ikehashi and Araki 1986, Chen *et al.*. 2008,  Yang *et al.*. 2012 |
| *S6* | 6 | RM3183-C133A | ♂/♀ | *O. rufipogon → O. sativa* | Sano 1989  Koide *et al*. 2008a |
| *S-7* | 7 | *Rc*-*Est-9* | ♀ | *indica → japonica* | Yanagihara *et al.*. 1992, Yu *et al.*. 2016 |
| *S-8* | 6 | Cat-1-Pox-5 | ♀ | *indica → japonica* | Wan *et al.* 1993 |
| *S-9* | 4 | RM185-RM3742 | ♀ | *indica → japonica* | Wan *et al.*. 1996 |
| *S10* | 6 | linked with *Wx* gene | ♂/♀ | *indica → japonica* | Sano *et al.* 1994b |
| *S11*(t) | 11 | linked with *la* gene | ♂ | *japonica → indica* | Sawamura and Sano 1996 |
| *S12*(t) |  |  | ♂ | *O. glumaepatula / O. sativa* | Sano 1994a |
| *S13* | 1 |  | ♂ | 1. *sativa / O. longistaminata* | Taneichi *et al*. 2005 |
| *S14*(t) |  |  | ♂ |  | Sano 1994a |
| *S15* | 12 | Pox2-Sdh1 | ♀ | *indica → japonica* | Wan *et al.*. 1996 |
| *S16* | 1 | hsp3-ch16 | ♀ | *japonica → indica* | Wan and Ikehashi. 1995 |
| *S17* | 12 | C2-C751 | ♀ | *japonica → indica* | Wan *et al.*. 1998 |
| *S18* | 10 | G1084-R1629 | ♂ | *O. glaberrima / japonica* | Doi *et al.*. 1998 |
| *S19* | 3 | RM523-RM14341 | ♂ | *O. glaberrima → japonica* | Taguchi *et al.*. 1999  Zhang *et al.* 2011b |
| *S20* | 7 | RM295-RM3831 | ♂ | *O. glaberrima → japonica* | Doi *et al.*. 1999 |
| *S21* | 7 | R1245-C213 | ♂ | *japonica → O. glaberrima* | Doi *et al.*. 1999 |
|  | 7 | RM5455-RM6063 | ♂ | *japonica → O. rufipogon* | Miyazaki *et al.*. 2007 |
| S22A and S22B | 2 | C1357(1.5cM) | ♂ | *japonica → O. glumaepatula* | Sobrizal *et al.*. 2000a,  Sakata *et al.* 2014 |
| *S23*(t) | 7 | R1789-C213 | ♂ | *japonica → O. glumaepatula* | Sobrizal *et al.*. 2000b  Fang *et al.*. 2019 |
| *S24*(t) | 5 | R830-R3166 | ♂ | *japonica → indica* | Kubo *et al.*. 2000 |
| *S25*(t) | 12 | G24-G189 | ♂ | *japonica → indica* | Kubo *et al.*. 2001 |
| *S26*(t) | 6 | L688-R2171 | ♀ | *japonica → indica* | Kubo and Yoshimura.. 2001 |
| *S27* | 8 | C347-L128 | ♂ | *japonica → O. glumaepatula* | Sobrizal and Yoshimura 2001, Yamagata *et al.*. 2010 |
|  | 8 | RM1309-RM8264 | ♂ | *japonica → O. nivara* | Win *et al.*. 2011 |
| *S28* | 4 | XNpb237(0.5cM) | ♂ | *O. glumaepatula → japonica* | Sobrizal and Yoshimura 2002, Yamagata *et al.*. 2010 |
| *S29*(t) | 2 | RM7033-RM7562 | ♂ | *O. glaberrima / japonica* | Hu *et al.* 2004 |
| *S29*(t) | 2 | RM8255-RM425 | ♀ | *japonica → indica* | Zhu *et al.* 2005b |
| *S30*(t) | 7 | RM11-RM 432 | ♀ | *indica → japonica* | Zhu *et al.* 2005a |
| *S31*(t) | 5 | RM5586-RM13 | ♀ | *indica → japonica* | Zhao *et al.* 2006 |
| *S32*(t) | 2 | RM236-RM211 | ♀ | *indica → japonica* | Li *et al.* 2005 |
| *S33*(t) | 1 | RM81-RM231 | ♂/♀ | *O. glaberrima → japonica* | Ren *et al.* 2005 |
| *S33*(t) | 3 | RM15621-RM15627 | ♂ | *japonica → indica* | Jing *et al.* 2007 |
| *S34*(t) | 11 | RM167-RM552 | ♂ | *japonica → indica* |  |
| *S34*(t) | 3 | RM251-RM231 | ♂ | *O. glaberrima → japonica* | Zhang *et al.* 2005 |
| *S35*(t) | 12 | RM19-RM6269 | ♀ | *indica → japonica* | Chen *et al.* 2012 |
| *S35* | 1 | RM6324-RM8105 | ♂ | *japonica → indica* | Kubo *et al.* 2008 |
| *S36* | 12 | M1-S36-RM3483 | ♂ | *O.nivara → japonica* | Win *et al.* 2009 |
| *S37* | 1 | RM466-RM113 | ♂/♀ | *O. glaberrima → japonica* | Xu *et al.* 2014 |
| *S38* | 4 | RM16251-RM16260 | ♂ | *O. glaberrima → japonica* |  |
| *S39* | 12 | RM5568-RM7582 | ♂ | *O. glaberrima → japonica* |  |
| *S40* | 1 | RM6289-HLY11 | ♂/♀ | *O. longistaminata → indica* | Chen *et al*. 2017 |
| *S44* | 6 | RM5814-RM20695 | ♂ | *O. longistaminata → indica* | Zhao *et al*. 2012 |
| *S51* | 1 | RM5-RM488 | ♂ | *O.meridionalis → japonica* | Li *et al.* 2018 |
| *S52* | 2 | RM6247-RM8 | ♂ | *japonica → O.meridionalis* |  |
| *S53* | 2 | OSR17-RM110 | ♂ | *japonica → O.meridionalis* |  |
| *S54* | 7 | RM1093-RM7454 | ♂ | *O.meridionalis → japonica* |  |
| *S55/qHMS7* | 7 | RM234-RM5623 | ♂ | *japonica → O.meridionalis* | Li *et al.* 2018,  Yu *et al.* 2018 |
| *S56* | 7 | RM20797-RM1093 | ♂ | *O. glumaepatula → japonica* | Zhang *et al.* 2018 |
| *Sa* | 1 | CDO568(6.4cM) | ♂ | *indica → japonica* | Zhuang *et al.* 1999,  Long *et al.* 2008 |
| *Sb* | 5 | PSM8-PSM202 | ♂ | *indica → japonica* | Li *et al.*. 2006 |
| *Sc* | 3 | RG227STS-RM218 | ♂ | *indica → japonica* | Zhang *et al*. 2001,  Shen *et al.* 2017 |
| *Sd* | 1 | PSM13-RM283 | ♂ | *indica → japonica* | Zhang *et al.* 1994  Li 2003 |
| *Se* | 12 | PSM180-PSM182 | ♂ | *indica → japonica* | Zhang *et al*. 1994  Zhu *et al.* 2008 |
| *Sf* |  |  | ♂ | *indica → japonica* | Zhang *et al.* 1994 |
| *S-p* | 11 | G24 | ♀ | *indica → japonica* | Zhu *et al.* 1998 |
| *hsa1* | 12 | *SP-5229-SP-5232* | ♀ | *japonica → indica* | Kubo *et al*. 2016 |
| *f5-Du* | 5 | WFPM3-WFPM5 | ♂ | *indica → japonica* | Wang *et al*. 1998, 2006 |
| *qS12* | 12 | MS062-MS102 | ♂ | *indica → japonica* | Zhang *et al*. 2011a |
| *DPL1* | 1 | S11214 | ♂ | *indica / japonica* | Mizuta *et al.* 2010, |
| *DPL2* | 6 | S1520 |  |  |  |
| *DGS1* | 4 | RM471-RM1359 | ♂ | *japonica / O. nivara* | Nguyen *et al*. 2017 |
| *DGS2* | 7 | RM6652-RM1353 |  |  |  |
